# The complete chloroplast genome of *Typha angustifolia* and its phylogenetic position in Typhaceae

**DOI:** 10.1080/23802359.2024.2389913

**Published:** 2024-08-09

**Authors:** Weiwei Lv, Jiayi Qian, Qian Long, Songting Wang

**Affiliations:** a Key Laboratory of Plant Stress Biology, School of Life Sciences, Henan University, Kaifeng, China; b College of Life Sciences, Henan Agricultural University, Zhengzhou, China

**Keywords:** *Typha angustifolia*, Typhaceae, chloroplast genome, phylogeny inference

## Abstract

*Typha angustifolia*, commonly known as narrowleaf cattail, is a marginal, semi-aquatic, herbaceous perennial species with both ecological and edible values. In this study, the complete chloroplast (cp) genome of *T. angustifolia* was assembled using the next-generation sequencing technology. The whole cp genome was 161,597 bp in length, consisting of a large single copy (LSC, 89,119 bp) and a small single copy (SSC, 18,550 bp) separated by two copies of inverted region (IR, 26,964 bp). The genome encoded 113 unique genes, including 79 protein-coding genes, 30 tRNA genes, four rRNA genes, with 19 duplicated genes in the IR regions. Phylogenetic analysis showed that *T. angustifolia* is sister to *Typha orientalis* in the family Typhaceae. The cp genome of *T. angustifolia* is reported for the first time, which will provide essential and important genetic resources for future phylogenetic investigation within the genus *Typha*.

## Introduction

*Typha angustifolia* L. 1753, commonly known as lesser reedmace or narrowleaf cattail, is widely distributed in shallow water of lakes, ponds, and rivers in Southwest Asia, Australia, Europe, and North America (Sun and Simpson 2010). The *T. angustifolia* plants can provide nesting areas and covers for many species of wetland animals and their leaves can proved nesting materials for various species of wetland birds including blackbirds and marsh wrens, thereby is known for attracting wildlife (Petty 2005). Meanwhile, the peeled rhizomes of *T. angustifolia* can be cooked and eaten like potatoes, the young spring shoots can be used as a substitute for asparagus, and the young immature flowers can be boiled and eaten (Plaisted 2006). The pollen of *T. angustifolia*, also known as Puhuang, has been used as a traditional herbal medicine with the ability to improve microcirculation and relieve pain (Ma et al. 2011; Shi et al. 2014).

Chloroplast DNA (cpDNA), which is maternally inherited in most angiosperms, has widely been used for tracing demographic history (Thomson et al. 2010) and inferring phylogenetic relationships (Wang et al. 2024). In the genus *Typha*, there were 42 recognized species all over the world (Govaerts et al. 2021). However, the cp genomes of only six species have been successfully assembled and published. In this study, the cp genome of *T. angustifolia* was firstly sequenced and annotated using the next-generation technology and the relationships between *T. angustifolia* and its closely related species in Typhaceae were investigated. Our results provide useful tools for future studies on the phylogeny, plastome evolution, and population genetics of *Typha*.

## Materials and methods

**Figure 1.**
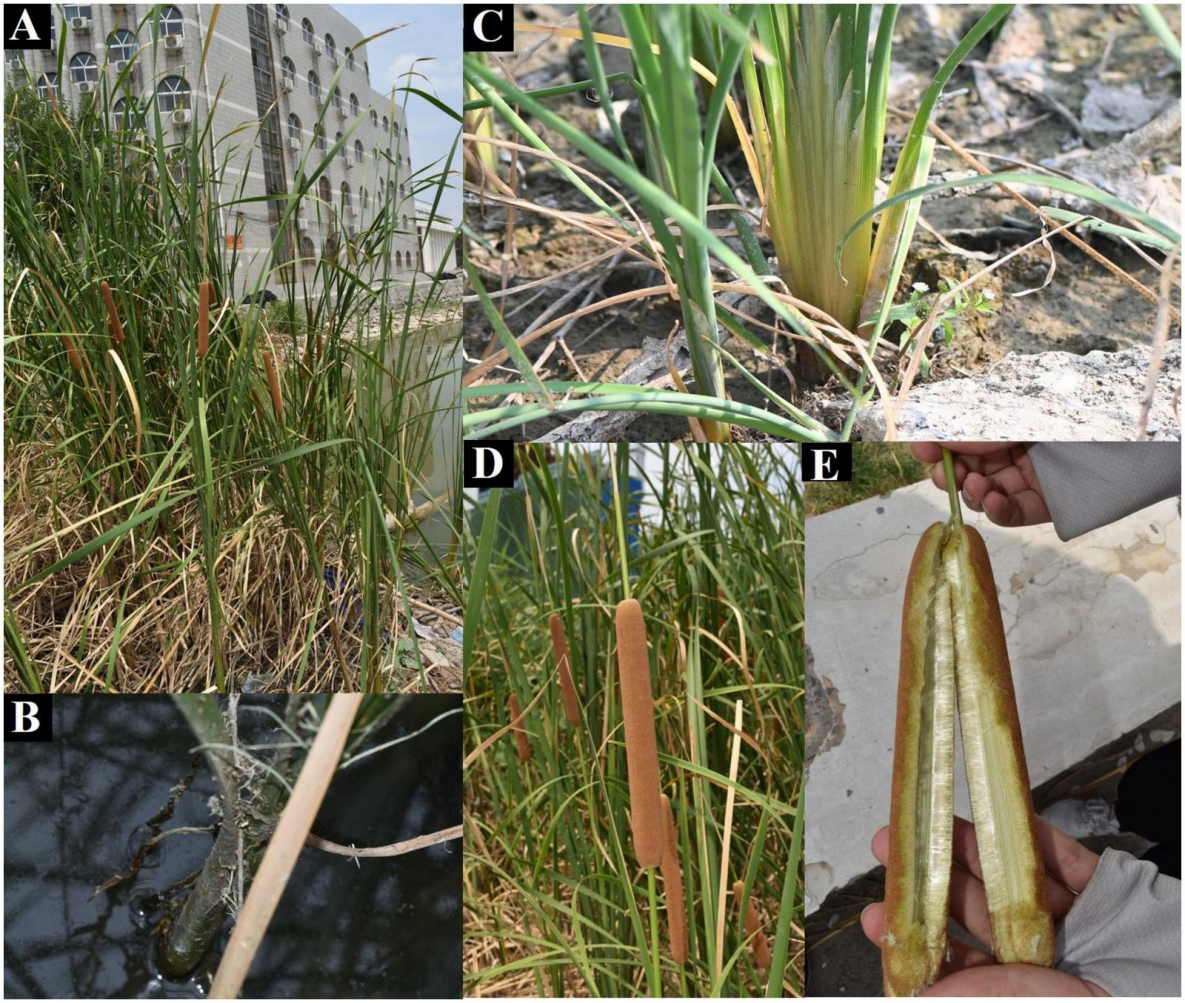
The morphological characteristics of *Typha angustifolia*, taken by Songting Wang in Henan University, Kaifeng, China. (A) Overall photo. *T. angustifolia* is a perennial, aquatic or bog herb. (B) Aerial stem. The aerial stem of the plant is very stout. (C) Sheath. The sheath of the plant embraces the stem. (D) Pistillate inflorescence. Female part of spikes (5-)15–30 cm. (E) Female flower. Female flowers with bracteoles filiform.

One individual of *T. angustifolia* was collected from Jinming Campus of Henan University (Kaifeng, China; 114°18′37.56″E, 34°49′20.96″N). The collected sample was neither privately owned nor protected and the field study did not involve endangered or protected species, therefore no specie permission was required. The plant was identified as *T. angustifolia* by Dr. Songting Wang (Figure 1), a voucher specimen (*WST20240501; Songting Wang,* wsthenu@126.com) was deposited at the Herbarium of Henan University (HHN).

Fresh leaves were collected from the plant and the total genomic DNA was extracted using Plant DNAzol Reagent (LifeFeng, Shanghai) according to the manufacturer’s protocol. High-quality DNA was sheared and sequenced on Illumina HiSeq X10 by Beijing Genomics Institute (BGI, Wuhan, China) with 150 bp paired-end reads. The raw reads were first trimmed using the CLC Genomics Workbench (CLC Inc. Aarhus, Denmark). The parameters set in CLC were as follows: 0.001 for trim using quality scores, 2 for trim ambiguous nucleotides, 50 bp for discard reads below length. We assembled the complete chloroplast genome using GetOrganelle v 1.7.1 (Jin et al. 2020) with the parameters ‘-R 30 -k 21,45,65,85,105 -F embplant_pt’. The genome was annotated in Geneious R11 (Kearse et al. 2012), and putative starts, stops, and intron positions of each gene were corrected by comparison with the homologous gene in the cp genome of *Typha przewalskii* Skvortsov 1943 (GenBank accession number: OK539747). The map of the genome was drawn using CPGView (Liu S et al. 2023), and the genome sequence was deposited into GenBank with the accession number PP790750.

To identify the phylogenetic position of *T. angustifolia*, phylogenetic tree for 25 cp genome sequences of Typhaceae was reconstructed using RAxML-HPC v8.1.11 (Stamatakis 2006) on the CIPRES cluster (Miller et al. 2010) with two *Brocchinia* species as the outgroup. PARTITIONFINDER v2.1.1 (Lanfear et al. 2017) was used to determine the optimal nucleotide substitution models (GTR + I + G) for the cp genome sequences.

## Results

**Figure 2.**
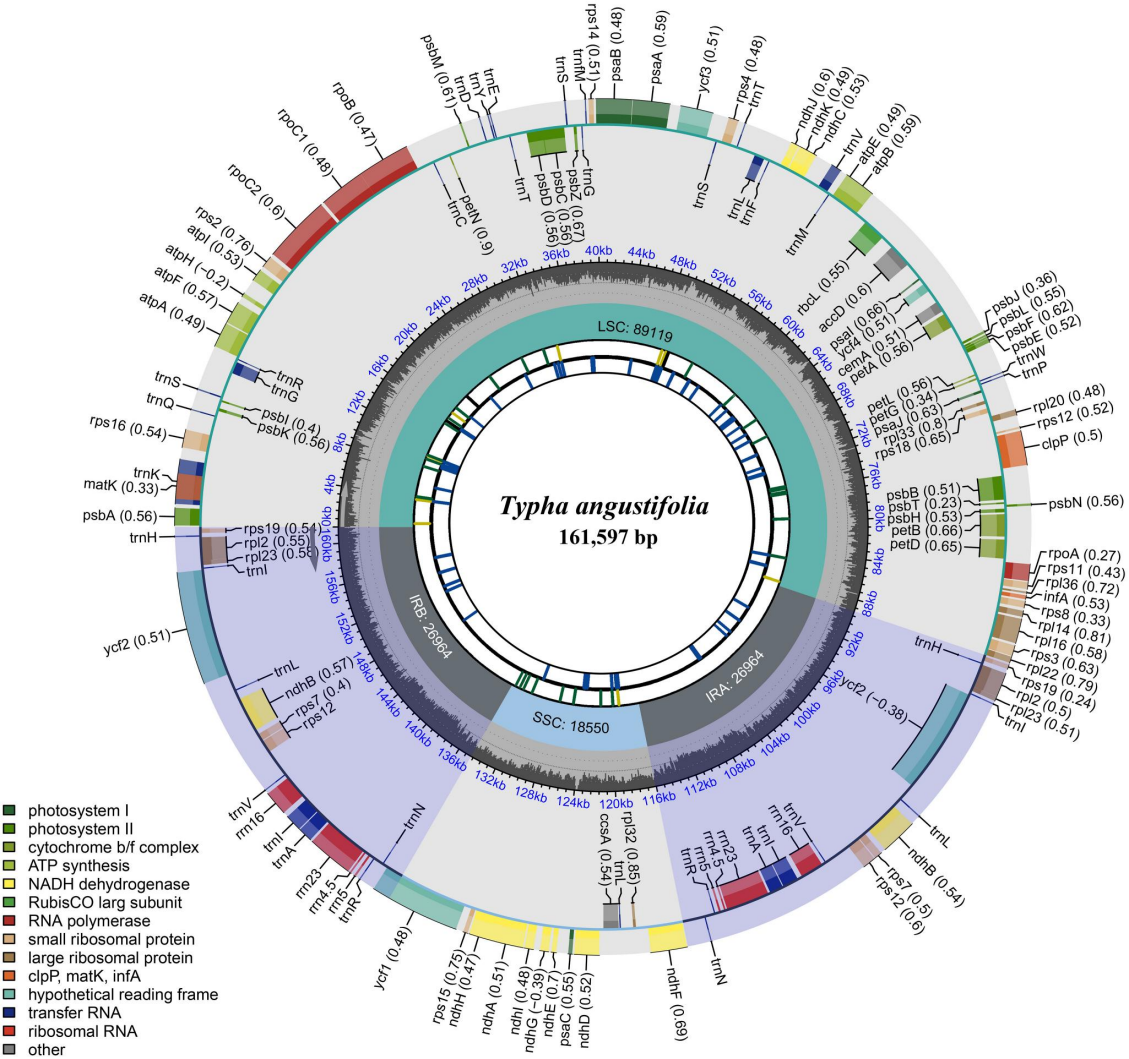
Graphic representation of features identified in the chloroplast genome of *Typha angustifolia* using CPGView. The map contains five tracks, from the center to the outside: (1) long tandem repeats as short blue bars, (2) short tandem repeats or microsatellite sequences as short bars with different colors, (3) size of the LSC, SSC, and IR regions, (4) GC content, and (5) genes having different colors based on their functional groups, colored according to the legend.

**Figure 3.**
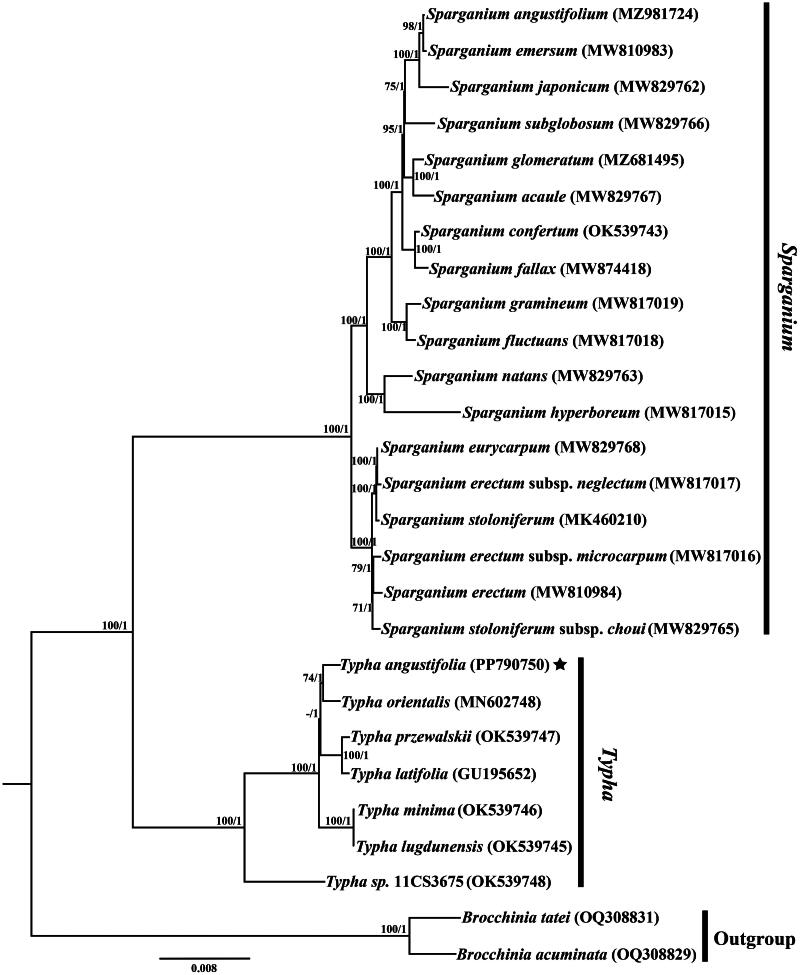
Phylogenetic tree reconstruction of Typhaceae using Bayesian inference (BI) analysis based on whole chloroplast genome sequences. Numbers above the lines represent ML bootstrap/Bayesian posterior probability (ML/BI) values. ML bootstrap values ≤ 50% are indicated by the dash. GenBank accession numbers of taxa are shown after the species name. The following sequences were used: *Sparganium angustifolium* (MZ981724, You et al. 2022), *S. emersum* (MW810983, direct submission), *S. japonicum* (MW829762, Zhang et al. 2022), *S. subglobosum* (MW829766, Zhang et al. 2022), *S. glomeratum* (MZ681495, Lu QX et al. 2021), *S. acaule* (MW829767, Zhang et al. 2022), *S. confertum* (OK539743, Wu et al. 2022), *S. fallax* (MW874418, Zhang et al. 2021), *S. gramineum* (MW817019, Zhang et al. 2022), *S. fluctuans* (MW817018, Zhang et al. 2022), *S. natans* (MW829763, Zhang et al. 2022), *S. hyperboreum* (MW817015, Zhang et al. 2022), *S. eurycarpum* (MW829768, Zhang et al. 2022), *S. erectum* subsp*. neglectum* (MW817017, Zhang et al. 2022), *S. stoloniferum* (MK460210, Su et al. 2019), S. *erectum* subsp. *microcarpum* (MW829765, Zhang et al. 2022), *Typha orientalis* (MN602748, Liu et al. 2020), *T. przewalskii* (OK539747, Wu et al. 2022), *T. latifolia* (GU195652, Jansen et al. 2007), *T. minima* (OK539746, Wu et al. 2022), *T. lugdunensis* (OK539745, Wu et al. 2022), *T.* sp. 11CS3675 (OK539748, Wu et al. 2022), *Brocchinia tatei* (OQ308831, direct submission), *B. acuminata* (OQ308829, direct submission).

We generated 37,535,325 paired-end reads for *T. angustifolia*, and 3,501,529 reads were removed from the raw data after trimming low-quality sequences. The minimal and average read mapping depths for assembled genome were 68× and 180× (Figure S1). The cp genome of *T. angustifolia* assembled in this study was 161,597 bp in length (Figure 2), exhibiting a typical quadripartite structure comprised of a pair of inverted repeat regions (IR, 26,964 bp), one large single-copy region (LSC, 89,119 bp) and one small single-copy region (SSC, 18,550 bp). The genome encoded 113 unique genes, including 79 protein-coding genes, 30 tRNA genes, four rRNA genes, with 19 duplicated genes in the IR regions. Additionally, six tRNA genes and nine protein-coding genes contained single intron, *clp*P and *ycf*3 contained two introns (Figure S2), and *rps*12 was detected as a trans-splicing gene (Figure S3). Phylogenetic analysis showed that all the *Typha* and *Sparganium* species formed a monophyletic clade, and *T. angustifolia* has a close relationship with *Typha orientalis* C. Presl 1851 in the family Typhaceae (Figure 3).

## Discussion and conclusions

In *Typha*, the availability of cp genomes remained relatively limited and only six species has been sequenced. Here, we assembled and annotated the cp genome of *T. angustifolia*. The cp genome has a circular and typical quadripartite structure, which is consistent with most other angiosperms (Lu RS et al. 2016; Wang et al. 2024). Comparative analysis showed that the cp genomes of *Typha* were relatively conserved, there were no rearrangement occurred in gene organization (Figure S4), and the genome size of *Typha* was slightly ranged from 160,969 bp (*T. orientalis*; Liu ZD et al. 2020) to 161,614 bp (*T. przewalskii*; Wu et al. 2022).

Previous phylogenetic studies of *Typha* have focused on a small number of molecular markers and different sample sizes, and the results were often controversial. Kim and Choi (2011) showed a monophyly of *T. angustifolia*, but the relationship position within the genus *Typha* was not resolved in the plastid gene tree. Zhou et al. (2018) investigated the phylogeny of *Typha* based on seven cp DNA regions and revealed a non-monophyly of *T. angustifolia*. In this study, the *Sparganium* and *Typha* species were first divided into two branches on the phylogenetic tree (Figure 3), and *T. angustifolia* showed a sister relationship with *T. orientalis* in the genus *Typha*.

## Supplementary Material

Supplementary materials.docx

## Data Availability

The genome sequence data that support the findings of this study are openly available in GenBank of NCBI at https://www.ncbi.nlm.nih.gov/ under the accession no. PP790750. The associated Bio-Project, SRA and Bio-Sample numbers of the raw sequence data for assembling the cp genome are PRJNA1112744, SRR29063669, and SAMN41432773, respectively.
