# Supplementary material for: The complete chloroplast genome of Typha angustifolia and its phylogenetic position in Typhaceae
Source: Mitochondrial DNA B Resour. 2024 Aug 9;9(8):1034–8. doi: 10.1080/23802359.2024.2389913 (PMC11318489; doi:10.1080/23802359.2024.2389913)
Supplement: Supplementary materials.docx [file TMDN_A_2389913_SM0495.docx]

Supplemental materials


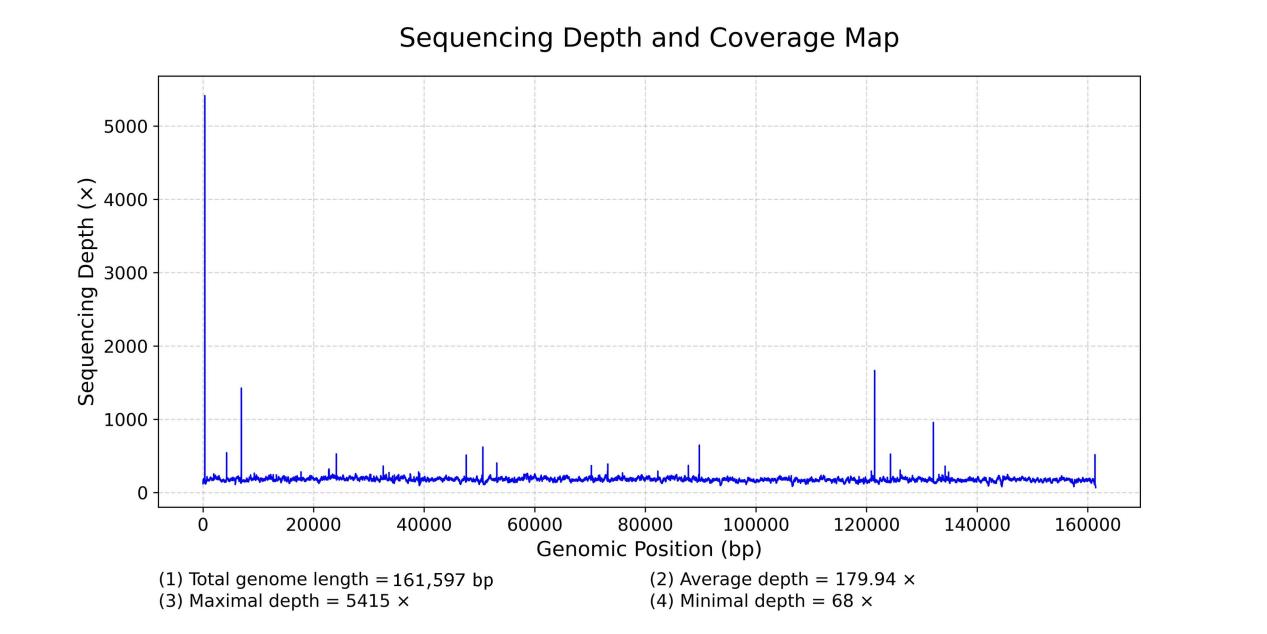


Figure S1. Sequencing depth and coverage map of the *Typha angustifolia* chloroplast genome. This plot shows the sequencing reads coverage and depth of *Typha angustifolia* chloroplast genome, confirming the assembly of the genome. The minimal and average read mapping depths for assembled genomes were 68× and 179.94×, respectively.


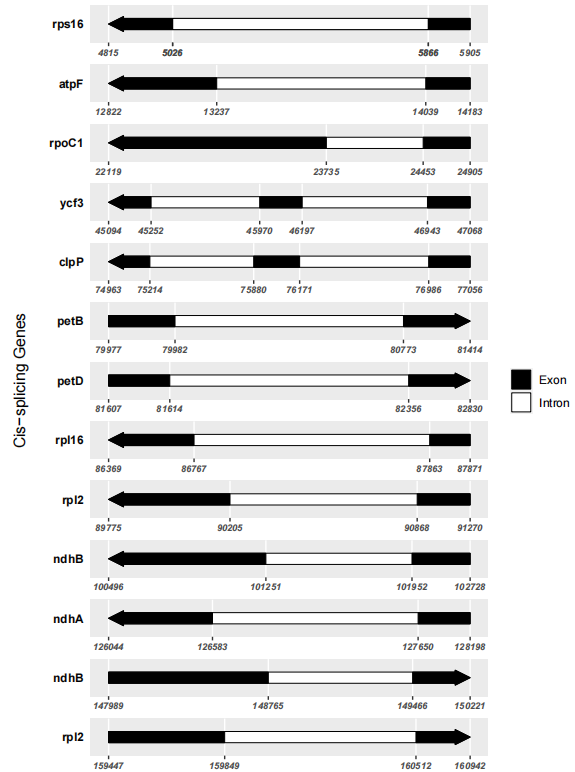


Figure S2. Schematic map of the cis-splicing genes in the chloroplast genome of *Typha angustifolia*. In the diagram, cis-splicing exons are shown in black, and introns are shown in white. The arrows indicate the sense direction of transcription. The numbers within the figure denote the location in the chloroplast genome of *Typha angustifolia*. Note that the lengths of exons and introns are not drawn to scale. This map was generated with CPGView.


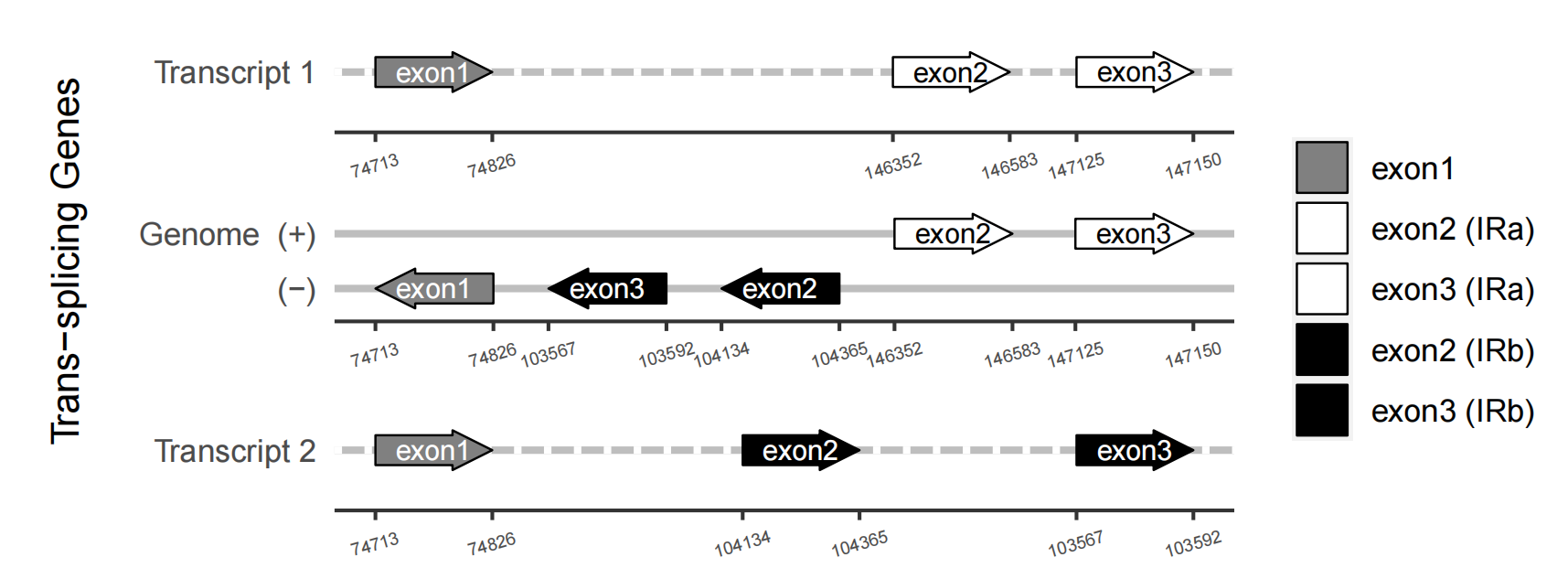


Figure S3. Schematic map of the trans-splicing gene *rps*12 in the chloroplast genome of *Typha angustifolia*. The arrows indicate the sense direction of transcription. The numbers in the figure represent the location in the chloroplast genome of *Typha angustifolia*. This map was generated with CPGView.


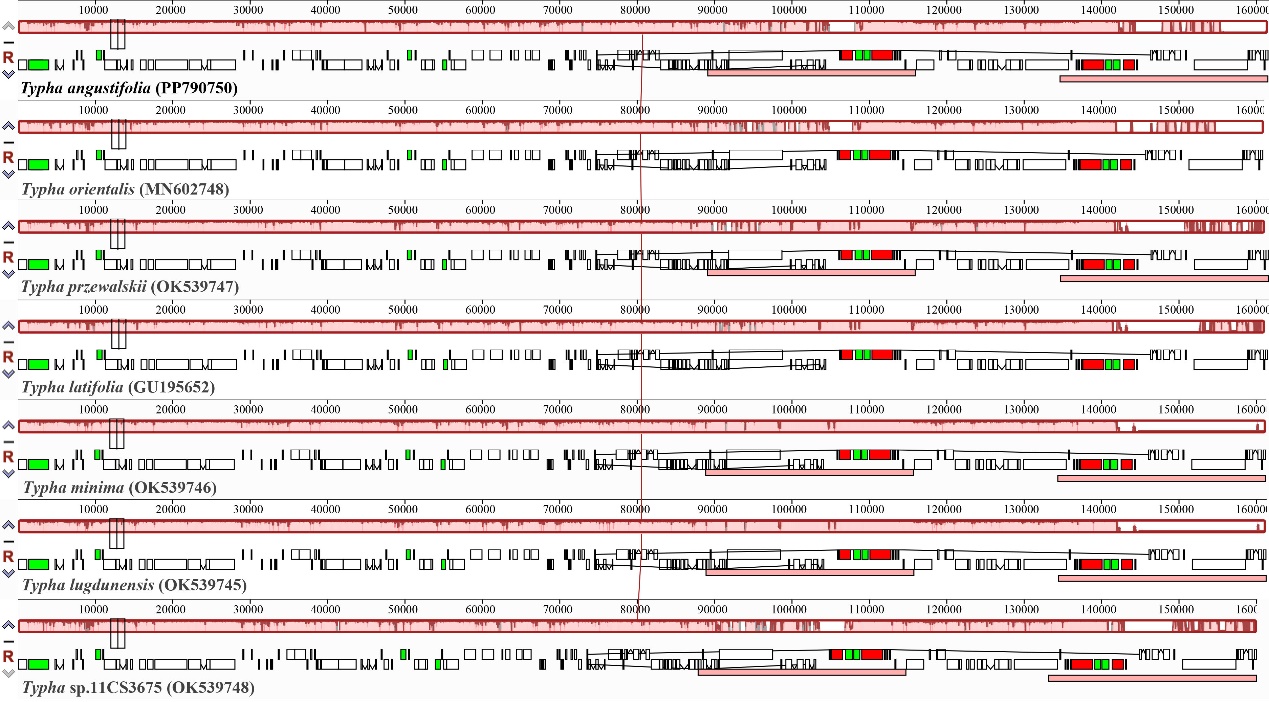


Figure S4. Mauve alignment of seven complete chloroplast genomes of *Typha* species. Within each of the alignment, local collinear blocks are represented by blocks of the same color connected by lines.
